# Association between dietary intake of flavonoids and hyperuricemia: a cross-sectional study

**DOI:** 10.1186/s12889-023-16134-4

**Published:** 2023-06-24

**Authors:** Houlin Li, Lin Shi, Xuelan Chen, Mo Wang

**Affiliations:** Department of Nephrology, Children’s Hospital of Chongqing Medical University, Ministry of Education Key Laboratory of Child Development and Disorders, National Clinical Research Center for Child Health and Disorders, China International Science and Technology Cooperation Base of Child Development and Critical Disorders, Chongqing Key Laboratory of Pediatrics, No. 136, Zhongshan Er Road, Yuzhong District, Chongqing, 400014 China

**Keywords:** Flavonoids, Uric acid, Hyperuricemia, NHANES

## Abstract

**Background:**

Previous research has demonstrated flavonoid intake was closely related to hyperuricemia. The purpose of this study was to examine whether flavonoid intake was associated with serum uric acid and hyperuricemia in U.S. adults.

**Methods:**

The study sample consisted of 8,760 participants enrolled in the National Health and Nutrition Examination Survey (NHANES) from 2007 to 2010. Flavonoid consumption was measured using a two-day recall questionnaire on dietary intake. Hyperuricemia was defined based on the serum uric acid levels, determined as ≥ 7 mg/dL for males and ≥ 6 mg/dL for females. The study utilized multivariate linear regression to determine the correlation between flavonoid consumption and serum uric acid levels. Additionally, analyses involving multivariate logistic regression and restricted cubic splines (RCS) were conducted to evaluate the potential link between flavonoid consumption and hyperuricemia. All analyses were adjusted for possible confounding variables.

**Results:**

The study revealed a negative correlation between serum uric acid levels and elevated levels of anthocyanidins and flavanones, with significant p-trends of < 0.001 and 0.02 respectively. The multivariate analysis showed that anthocyanidins and flavanones intake had a significant negative association with the risk of hyperuricemia, with p-trend value being < 0.001 and 0.01, respectively. Flavan-3-ols, flavonols, and all flavonoids exhibited a non-linear association with the incidence of hyperuricemia, with significant p-nonlinear values of < 0.001, 0.04, and 0.01 respectively.

**Conclusion:**

Our study demonstrated that individuals who follow a diet rich in anthocyanins and flavanones had significantly lower serum uric acid levels and a lower incidence of hyperuricemia.

**Supplementary Information:**

The online version contains supplementary material available at 10.1186/s12889-023-16134-4.

## Introduction

Uric acid is the ultimate product of purine nucleotide metabolism. When uric acid levels are beyond a certain threshold, hyperuricemia develops. Hyperuricemia is widely recognized as a risk factor for various chronic diseases, such as gout [1], cardiovascular disease [2], type 2 diabetes [3], hypertension [4], chronic kidney disease [5], and obesity [6]. Over the past few decades, the incidence of hyperuricemia has risen significantly [7], posing a serious threat to public health. The incidence of hyperuricemia varies among different populations and geographic regions, and several cross-sectional studies have reported its prevalence to range from 8.4% to 25.8% [8–11]. Therefore, identifying potential preventive factors against an elevation in serum uric acid concentration is of utmost importance.

Flavonoids are highly abundant polyphenolic phytochemicals present in many plant-based foods [12]. Anthocyanidins, flavan-3-ols, flavanones, flavones, flavonols, and isoflavones are the main subclasses of flavonoids. Due to their antioxidant, anti-inflammatory, immune-regulating, anticancer, and anti-proliferative properties, flavonoids have been suggested to possess an inverse association with various chronic illnesses [13, 14]. Several flavonoids have the potential to alleviate hyperuricemia by increasing uric acid excretion, reducing uric acid reabsorption, inflammation, and xanthine oxidase activity [15–18]. Although these studies showed a negative association between flavonoids and hyperuricemia, their scope was largely limited to certain components and experimental conditions. A comprehensive evaluation of the association between daily dietary flavonoid intake and hyperuricemia is deemed necessary.

This study assessed the correlation between flavonoid intake, and serum uric acid level and hyperuricemia in U.S. adults by utilizing flavonoid intake data provided by the United States Department of Agriculture (USDA), combined with sociodemographic and laboratory data from the National Health and Nutrition Examination Survey (NHANES) from 2007 to 2010.

## Methods

### Study population

NHANES is a cross-sectional program that is conducted nationally by the National Center for Health Statistics (NCHS). Its purpose is to evaluate the health and nutritional status of the U.S. population using a stratified multistage sample methodology, and it has been approved by the NCHS Ethics Review Board. The data collection process for NHANES involved an in-home interview, a health examination conducted in a mobile examination center (MEC), and a follow-up telephone interview. We used publicly available data from NHANES 2007–2010. All participants provided their written informed consent.

**Fig. 1:**
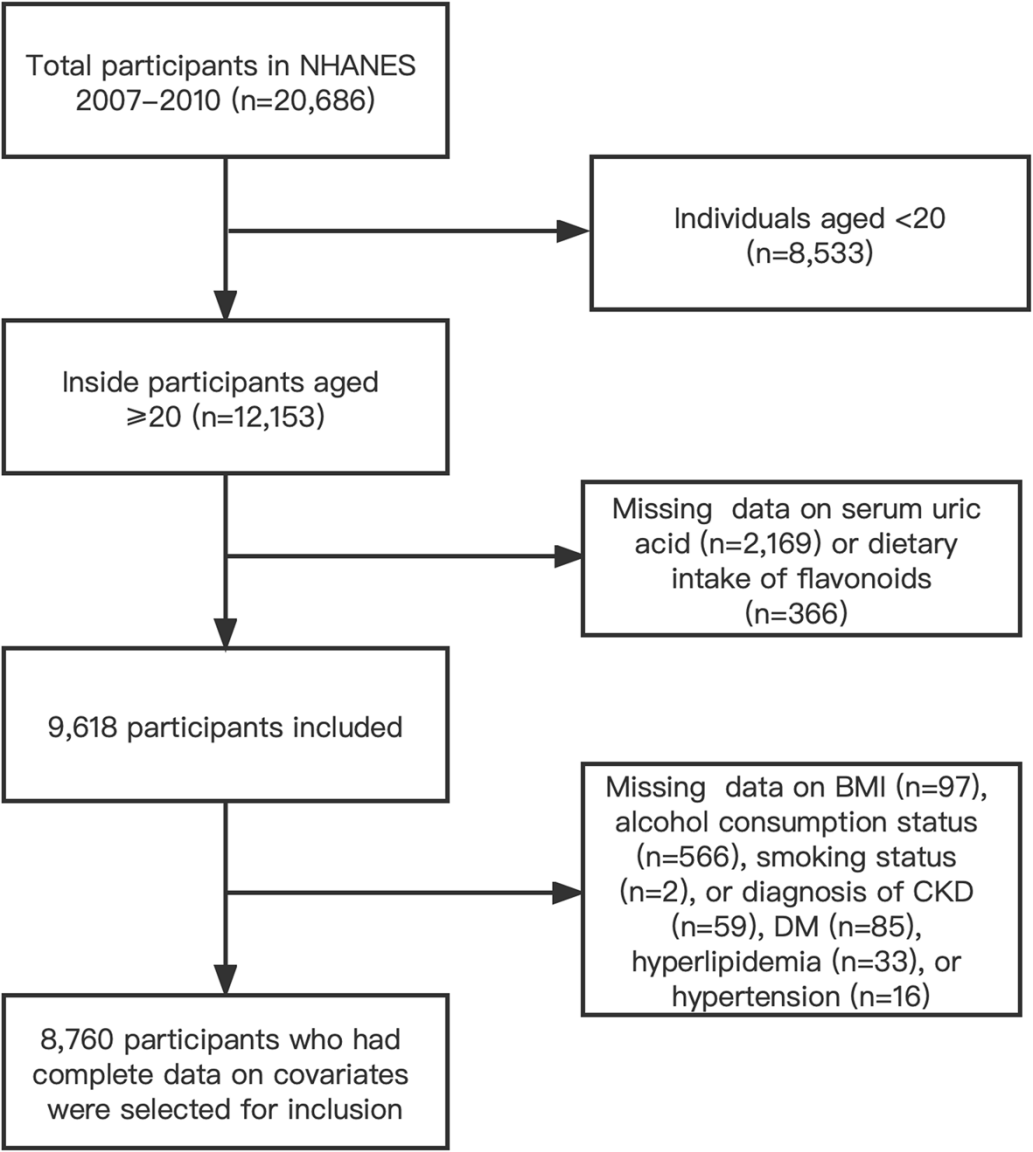
Sample inclusion flow diagram

There were data on flavonoid intake and serum uric acid for a total of 9618 participants older than 20 years old. Of these, we eliminated 858 participants who lacked information regarding their body mass index (BMI), alcohol consumption, smoking status, hyperlipidemia, chronic kidney disease (CKD), diabetes mellitus (DM), and hypertension. Finally, 8,760 participants with complete covariates were selected for inclusion (Fig. 1).

## Serum uric acid and hyperuricemia

The NHANES researchers measured serum uric acid concentrations in a MEC. Hyperuricemia was defined as serum uric acid levels of ≥ 7 mg/dl for males and ≥ 6 mg/dl for females [19].

## The intake of flavonoids

The Flavonoid Database for the USDA Food Codes 2007–2010 contains flavonoid values for all foods/beverages in version 5.0 of the USDA Food and Nutrient Database for Dietary Studies (FNDDS). The Flavonoid Database includes the levels of 29 flavonoids in 6 flavonoid classes (Table S1) to match the appropriate NHANES 2007–2010 dietary data release. The mean daily intake of flavonoids (measured in milligrams per 100 g of foods and beverages) during the NHANES 2007–2010 was estimated through a two-day dietary recall. The two-day dietary recall documented a participant's food intake for two days using a face-to-face interview and a subsequent telephone conversation about a week later to obtain further information.

## Covariates

The NHANES researchers collected information about participants’ age (years), gender (male, female), race/ethnicity (White, Black, Mexican- American, others), education (less than high school, completed high school or more than high school), poverty status (yes or no), alcohol consumption (no, mild, moderate, and heavy) and smoking status (never, former, and now) through a structured questionnaire. Poverty status was defined by the income poverty income ratio (PIR) of < 1 (below the poverty threshold) versus ≥ 1 (reference) [20]. Alcohol consumption was classified as follows: 1) no consumption (individuals who had not consumed any alcohol in the past year or had consumed less than 12 drinks in their lifetime), 2) heavy consumption (≥ 3 drinks per day for women, ≥ 4 drinks per day for men, or ≥ 5 binge drinking days per month), 3) moderate consumption (≥ 2 drinks per day for women, ≥ 3 drinks per day for men, or ≥ 2 binge drinking days per month), and 4) mild consumption (individuals who did not meet the criteria for the categories described above) [21]. Smoking status was categorized as follows: 1) never (individuals who had smoked less than 100 cigarettes in their lifetime), 2) former (individuals who had smoked at least 100 cigarettes in their lifetime, but were currently non-smokers), and 3) now (individuals who had smoked at least 100 cigarettes in their lifetime and were current smokers) [22]. The participant’s height and weight were measured during a physical examination, and body mass index (BMI) was calculated as weight in kilograms over height in meters squared (kg/m^2^). The BMI ≥ 25 indicated that participants were overweight. According to the Adult Treatment Panel III (ATP3), hyperlipidemia was determined as one of three conditions: 1) hypertriglyceridemia: triglycerides (TG) ≥ 150 mg/dl; 2) hypercholesterolemia: total cholesterol (TC) ≥ 200 mg/dl or low-density lipoprotein (LDL) ≥ 130 mg/dl or high-density lipoprotein (HDL) ≤ 40 mg/dl in males, ≤ 50 mg/dl in females; 3) use of lipid-lowering drugs [23]. CKD was determined as eGFR < 60 mL/ min/1.73 m^2^, and/or albumin-to-creatinine ratio (ACR) > 30 mg/g. eGFR was calculated by the CKD-EPI formula [24]. The diagnostic criteria for DM are random blood glucose ≥ 11.1 mmol/l, fasting glucose ≥ 7.0 mmol/l, two-hour oral glucose tolerance test (OGTT) blood glucose ≥ 11.1 mmol/l, glycohemoglobin HBA1c > 6.5%, or reporting a previous diagnosis. The definition of Hypertension was resting blood pressure (BP) persistently at or above 140/90 mmHg or reporting a previous diagnosis.

## Statistical analysis

For all statistical calculations, sampling weights (wtmec2yr) provided by the NHANES were applied in R 4.2.1 which adequately accounted for the stratification and complexity of NHANES sampling. In this study, the 8,760 participants were weighted to approximate a population of 167,288,047. First, continuous variables were shown as weighted means (standard errors), and categorical variables were displayed as unweighted numbers (weighted percentages).

Next, the associations between flavonoid intake and serum uric acid were then estimated using linear regression. The intake of flavonoid subclass and total flavonoids was categorized into five quintiles (Table S2) and analyzed using generalized linear regression models with the low–intake group as the reference group. In the regression models, tests for trend (p–trend) were undertaken across quintiles utilizing the median of these flavonoids in each quartile as a linear variable. In addition, ln-transformed flavonoids that had been transformed via the natural logarithm also were utilized as continuous variables for linear regression. Each component of flavonoids was the only independent variable in Model 1, and Model 2 was adjusted for age, sex, race, poverty status, education, smoking status, alcohol consumption, and BMI. Based on Model 2, Model 3 was further adjusted for hyperlipidemia, CKD, DM, and hypertension.

Then, logistic regression was utilized to estimate prevalence odds ratios (ORs) and 95% confidence intervals (CIs) as a cross-sectional assessment of correlations between flavonoids and hyperuricemia. In the previously mentioned models, five quintiles of flavonoid subclasses and total flavonoids were included as categorical variables, and ln-transported flavonoids were included as continuous variables. Furthermore, restricted cubic splines (RCS) were employed in logistic regression Model 3 to investigate the potential non-linear correlations between flavonoids and the probabilities of hyperuricemia. The *p*-values for nonlinear trends were computed using Wald testing for RCS coefficients.

## Results

Table 1 presents the participants’ weighted sociodemographic characteristics. The study involved 8,760 participants who took part in the NHANES 2007–2010 survey, including 4,392 men and 4,332 women. The selected subjects from the NHANES 2007–2010 had an average age of 47.01 years, with White being the predominant race/ethnicity (71.92%). 13.50% of individuals were living in poverty. 81.50% of participants have attained a high school diploma or higher. About half of the individuals (53.56%) had never smoked, and 32.29% of participants had never drunk. 19.66% of the participants were reported to have hyperuricemia. Participants diagnosed with hyperuricemia were found to have a markedly higher BMI and a higher incidence of other conditions including hyperlipidemia, hypertension, CKD, and DM. Additionally, Table 1 presents data on the dietary intake of flavonoids.

**Table 1:** Characteristics of participants included in NHANES 2007–2010 analyses (*n* = 8,760)

Variable	Total (*n* = 8,760)	Non-Hyperuricemia(*n* = 7,038, 80.34%)	Hyperuricemia (*n* = 1,722, 19.66%)
Age (years)	47.01(0.35)	46.11(0.35)	50.91(0.48)
Sex			
Male	4,392(50.14)	3,439(47.52)	953(57.22)
Female	4,368(49.86)	3,599(52.48)	769(42.78)
Race/ethnicity			
White	4,448(71.92)	3,544(71.68)	904(72.99)
Black	1,567(10.12)	1,179(9.75)	388(11.73)
Mexican–American	1,517(8.05)	1,292(8.41)	225(6.47)
Other	1,228(9.91)	1,023(10.16)	205(8.81)
Poverty status			
No	6,960(86.50)	5,581(86.35)	1,379(87.16)
Yes	1,800(13.50)	1,457(13.65)	343(12.84)
Education			
Less than high school	2,450(18.50)	1,970(18.53)	480(18.40)
Completed high school	2,077(23.73)	1,637(22.91)	440(27.36)
More than high school	4,233(57.76)	3,431(58.57)	802(54.24)
BMI			
< 25	2,457(30.81)	2,242(34.92)	215(12.79)
≥ 25	6,303(69.19)	4,796(65.08)	1,507(87.21)
Alcohol consumption			
Never	2,829(32.29)	2,227(26.06)	602(28.67)
Mild	2,762(31.53)	2,230(35.34)	532(33.94)
Moderate	1,285(14.67)	1,062(16.84)	223(14.29)
Heavy	1,884(21.51)	1,519(21.77)	365(23.10)
Smoking status			
Never	4,581(53.56)	3,744(54.06)	837(51.35)
Former	2,253(25.17)	1,682(23.83)	571(31.02)
Now	1,926(21.27)	1,612(22.10)	314(17.63)
Hyperlipidemia			
No	2,191(26.44)	1,915(28.71)	276(16.54)
Yes	6,569(73.56)	5,123(71.29)	1,446(83.46)
Chronic kidney disease			
No	7,211(86.73)	6,034(88.95)	1,177(76.99)
Yes	1,549(13.27)	1,004(11.05)	545(23.01)
Diabetes mellites			
No	7,141(86.51)	5,904(88.26)	1,237(78.86)
Yes	1,619(13.49)	1,134(11.74)	485(21.14)
Hypertension			
No	5,035(63.41)	4,414(67.91)	621(43.71)
Yes	3,725(36.59)	2,624(32.09)	1,101(56.29)
Uricacid (mg/dl)	5.49(0.03)	5.03(0.02)	7.51(0.03)
Isoflavones (mg)	1.68(0.18)	1.79(0.20)	1.21(0.23)
Anthocyanidins (mg)	12.84(0.96)	13.37(1.03)	10.56(1.39)
Flavan-3-ols (mg)	201.59(10.07)	193.61(9.18)	236.53(25.64)
Flavanones (mg)	13.16(0.51)	13.67(0.58)	10.94(0.78)
Flavones (mg)	0.92(0.04)	0.93(0.05)	0.87(0.04)
Flavonols (mg)	20.41(0.52)	20.11(0.52)	21.72(1.05)
All of flavonoids (mg)	250.61(10.67)	243.48(9.92)	281.82(26.51)

Continuous data were displayed as weighted means (standard errors), while categorical variables were exhibited as unweighted numbers (weighted percentages)

*BMI* Body mass index, *NHANES* The national health, and nutrition examination survey

Table 2 presents the results of linear regression analyses examining the associations between all flavonoids and flavonoid subclass intake levels and serum uric acid levels. In Model 2, a statistically significant negative correlation was observed between isoflavones and all flavonoid intake with serum uric acid (p-trend < 0.05). In all three models, there was a significant negative correlation seen between higher quintiles of anthocyanidins, and flavanones, and serum uric acid (p-trend < 0.05). In Model 3, participants in the 2^nd^, 3^rd^, 4^th^, and 5^th^ quintile of anthocyanidins (β (95% CI): -0.20(-0.34, -0.06), -0.12(-0.22, -0.02), -0.13(-0.25, -0.02), and -0.14(-0.24, -0.04), respectively), the 5^th^ quintile of flavanones (β (95% CI): -0.14(-0.23, -0.04)), had significantly lower serum uric acid compared with those in the reference quintiles. On the contrary, participants in the 4^th^ quintile of flavonols (β (95% CI): 0.12(0.04, 0.19)) had higher serum uric acid in Model 3. When substituting flavonoid values into linear regression models as continuous variables, it was discovered that ln-transformed anthocyanidins, and flavanones also exhibited similar negative associations with serum uric acid (β (95% CI): -0.03(-0.05, 0.00), -0.03(-0.05, -0.01), respectively) and ln-transformed flavonols also exhibited positive association with serum uric acid (β (95% CI): 0.04(0.01, 0.07).

**Table 2:** Associations between intake of flavonoid levels and serum uric acid, NHANES (2007–2010)

Variable	Serum Uric Acid β (95% CI)							
	Categorical variable						Continuous variable	
	Q1	Q2	Q3	Q4	Q5	p-trend	Ln-transformed	*p*-value
Isoflavones								
Model 1	Referent	/	-0.23(-0.39, -0.07)	-0.04(-0.12, 0.04)	-0.08(-0.18, 0.02)	0.05	-0.08(-0.13, -0.04)	** < 0.01**
Model 2	Referent	/	-0.24(-0.39, -0.10)	-0.06(-0.13, 0.00)	-0.09(-0.17, -0.01)	** < 0.01**	-0.05(-0.10, -0.01)	**0.03**
Model 3	Referent	/	-0.15(-0.30, 0.00)	-0.04(-0.11, 0.04)	-0.04(-0.13, 0.06)	0.10	-0.04(-0.08, 0.00)	0.08
Anthocyanidins								
Model 1	Referent	-0.23(-0.41, -0.04)	-0.20(-0.31, -0.09)	-0.21(-0.33, -0.09)	-0.32(-0.42, -0.22)	** < 0.001**	-0.07(-0.09, -0.04)	** < 0.001**
Model 2	Referent	-0.23(-0.39, -0.08)	-0.15(-0.25, -0.05)	-0.18(-0.30, -0.07)	-0.23(-0.33, -0.13)	** < 0.001**	-0.03(-0.06, -0.01)	**0.01**
Model 3	Referent	-0.20(-0.34, -0.06)	-0.12(-0.22, -0.02)	-0.13(-0.25, -0.02)	-0.14(-0.24, -0.04)	** < 0.001**	-0.03(-0.05, 0.00)	**0.04**
Flavan-3-ols								
Model 1	Referent	-0.02(-0.18,0.13)	-0.03(-0.16,0.09)	-0.12(-0.27,0.03)	0.01(-0.14,0.16)	0.71	0.00(-0.02,0.02)	0.88
Model 2	Referent	-0.12(-0.26, 0.01)	-0.12(-0.23, 0.00)	-0.23(-0.37, -0.08)	-0.08(-0.20, 0.05)	0.07	0.00(-0.02, 0.01)	0.77
Model 3	Referent	-0.08(-0.21, 0.06)	-0.05(-0.16, 0.07)	-0.12(-0.26, 0.01)	0.00(-0.11, 0.11)	0.81	0.00(-0.01, 0.02)	0.57
Flavanones								
Model 1	Referent	-0.22(-0.39, -0.04)	-0.09(-0.23, 0.04)	-0.13(-0.23, -0.03)	-0.20(-0.30, -0.10)	** < 0.001**	-0.04(-0.06, -0.02)	** < 0.01**
Model 2	Referent	-0.01(-0.20, 0.18)	-0.03(-0.14, 0.09)	-0.07(-0.17, 0.03)	-0.19(-0.28, -0.10)	** < 0.001**	-0.04(-0.05, -0.02)	** < 0.001**
Model 3	Referent	-0.05(-0.23, 0.14)	-0.01(-0.13, 0.10)	-0.04(-0.14, 0.06)	-0.14(-0.23, -0.04)	**0.02**	-0.03(-0.05, -0.01)	** < 0.01**
Flavones								
Model 1	Referent	0.00(-0.09, 0.08)	-0.06(-0.16, 0.03)	-0.10(-0.19, -0.01)	0.09(-0.03, 0.21)	0.40	0.05(-0.03,0.14)	0.22
Model 2	Referent	0.03(-0.06, 0.13)	-0.03(-0.13, 0.08)	-0.07(-0.16, 0.03)	0.01(-0.10, 0.12)	0.44	-0.03(-0.10, 0.05)	0.48
Model 3	Referent	0.06(-0.04, 0.16)	0.01(-0.11, 0.13)	-0.01(-0.10, 0.08)	0.06(-0.05, 0.16)	0.74	0.00(-0.07, 0.07)	0.97
Flavonols								
Model 1	Referent	-0.01(-0.14,0.11)	0.04(-0.08,0.16)	0.22(0.12,0.32)	0.24(0.12,0.37)	** < 0.001**	0.10(0.06,0.14)	** < 0.001**
Model 2	Referent	-0.05(-0.15, 0.04)	-0.04(-0.14, 0.06)	0.06(-0.01, 0.14)	0.03(-0.06, 0.12)	**0.02**	0.02(-0.01, 0.05)	0.21
Model 3	Referent	-0.02(-0.11, 0.07)	0.00(-0.10, 0.09)	0.12(0.04, 0.19)	0.07(-0.01, 0.16)	** < 0.01**	0.04(0.01, 0.07)	**0.03**
All of flavonoids								
Model 1	Referent	0.09(-0.05,0.22)	-0.03(-0.15,0.10)	-0.07(-0.16,0.03)	0.05(-0.09,0.19)	0.75	0.00(-0.02,0.03)	0.87
Model 2	Referent	-0.06(-0.16, 0.04)	-0.11(-0.22, 0.00)	-0.21(-0.32, -0.11)	-0.07(-0.18, 0.04)	**0.03**	-0.01(-0.03,0.01)	0.29
Model 3	Referent	-0.01(-0.10, 0.08)	-0.05(-0.16, 0.05)	-0.11(-0.20, -0.02)	0.01(-0.08, 0.10)	0.51	0.00(-0.02, 0.02)	0.93

The dietary intake of flavonoids was categorized into five quintiles and tests for trend (p–trend) based on variable containing the median value for each quintile. ln-transformed flavonoid also was utilized as continuous variables and *p*-value was used to test significance. Bold indicated statistically significant

Model 1 was a crude model with no adjusted covariates; Model 2 was adjusted for sex, age, race/ethnicity, poverty status, education, smoking status, alcohol consumption, and BMI; Model 3 was further adjusted for hyperlipidemia, CKD, DM, and hypertension based on Model 2

Table 3 presents the findings from multivariate logistic regression analyses examining the association between flavonoid intake levels and the risk of hyperuricemia. Statistically significant negative correlations were observed between higher quintiles of isoflavones and hyperuricemia in both Model 1 and Model 2 (p-trend < 0.05). Statistically significant negative associations were found between rising quintiles of anthocyanidins and flavanones intake levels with hyperuricemia across all three models (p-trend < 0.05). In Model 3, participants in the 4^th^ and 5^th^ quintile of anthocyanidins (OR (95%CI): 0.74(0.57,0.95), 0.70(0.53,0.90), respectively), the 5^th^ quintile of flavanones (OR (95%CI): 0.72(0.54,0.94)) had significantly lower odds of hyperuricemia compared with those in the reference quintiles. Ln-transformed anthocyanidins and flavanones also displayed similar negative associations with hyperuricemia in all models.

**Table 3:** Associations between intake of flavonoid levels and hyperuricemia, NHANES (2007–2010)

Variable	Hyperuricemia OR (95%CI)							
	Categorical variable						Continuous variable	
	Q1	Q2	Q3	Q4	Q5	p-trend	Ln-transformed	*p*-value
Isoflavones								
Model 1	Referent		0.63(0.43,0.92)	0.83(0.69,1.01)	0.80(0.65,0.98)	** < 0.01**	0.87(0.78,0.97)	**0.02**
Model 2	Referent	/	0.62(0.41,0.94)	0.82(0.67,1.02)	0.89(0.71,1.04)	** < 0.01**	0.92(0.82,1.03)	0.15
Model 3	Referent	/	0.64(0.42,0.98)	0.84(0.67,1.06)	0.91(0.71,1.15)	0.05	0.94(0.83,1.06)	0.27
Anthocyanidins								
Model 1	Referent	0.75(0.55,1.01)	0.85(0.70,1.02)	0.74(0.59,0.92)	0.68(0.54,0.85)	** < 0.01**	0.91(0.85,0.97)	** < 0.01**
Model 2	Referent	0.70(0.51,0.96)	0.81(0.67,0.99)	0.70(0.56,0.89)	0.64(0.52,0.89)	** < 0.001**	0.90(0.84,0.97)	**0.01**
Model 3	Referent	0.73(0.52,1.00)	0.85(0.68,1.05)	0.74(0.57,0.95)	0.70(0.53,0.90)	** < 0.001**	0.91(0.85,0.99)	**0.02**
Flavan-3-ols								
Model 1	Referent	0.86(0.67,1.12)	0.79(0.62,1.02)	0.80(0.59,1.07)	0.96(0.77,1.20)	0.65	1.01(0.97,1.04)	0.74
Model 2	Referent	0.84(0.64,1.10)	0.78(0.61,1.00)	0.78(0.57,1.07)	0.92(0.75,1.14)	0.23	1.00(0.98,1.04)	0.90
Model 3	Referent	0.86(0.64,1.16)	0.82(0.62,1.10)	0.84(0.59,1.21)	0.99(0.78,1.26)	0.95	1.01(0.98,1.05)	0.47
Flavanones								
Model 1	Referent	0.84(0.55,1.30)	0.87(0.72,1.06)	0.78(0.65,0.94)	0.73(0.59,0.90)	** < 0.01**	0.94(0.89,0.98)	**0.01**
Model 2	Referent	0.86(0.52,1.42)	0.87(0.69,1.09)	0.79(0.64,0.97)	0.70(0.55,0.90)	** < 0.01**	0.93(0.88,0.98)	**0.01**
Model 3	Referent	0.85(0.51,1.42)	0.89(0.70,1.13)	0.81(0.64,1.03)	0.72(0.54,0.94)	**0.01**	0.93(0.88,0.99)	**0.04**
Flavones								
Model 1	Referent	0.97(0.76,1.24)	0.86(0.69,1.09)	0.97(0.78,1.21)	0.96(0.76,1.21)	0.72	0.96(0.83,1.11)	0.56
Model 2	Referent	0.90(0.70,1.15)	0.81(0.62,1.03)	0.74(0.56,0.97)	0.91(0.70,1.09)	0.52	0.94(0.80,1.12)	0.53
Model 3	Referent	0.99(0.74,1.31)	0.86(0.64,1.15)	1.02(0.78,1.32)	0.97(0.73,1.30)	0.94	0.99(0.83,1.16)	0.88
Flavonols								
Model 1	Referent	0.91(0.75,1.11)	0.82(0.66,1.03)	1.07(0.89,1.29)	1.01(0.84,1.21)	0.35	1.02(0.96,1.09)	0.44
Model 2	Referent	0.90(0.74,1.09)	0.80(0.63,1.01)	1.01(0.83,1.21)	0.95(0.77,1.16)	0.88	1.01(0.94,1.07)	0.87
Model 3	Referent	0.93(0.76,1.15)	0.86(0.67,1.11)	1.14(0.92,1.41)	1.02(0.83,1.25)	0.25	1.03(0.96,1.10)	0.38
All of flavonoids								
Model 1	Referent	0.92(0.71,1.20)	0.83(0.66,1.05)	0.77(0.59,1.00)	0.95(0.78,1.15)	0.33	0.99(0.94,1.03)	0.51
Model 2	Referent	0.87(0.68,1.11)	0.78(0.62,0.99)	0.70(0.53,0.91)	0.86(0.70,1.05)	0.28	0.98(0.94,1.02)	0.31
Model 3	Referent	0.91(0.70,1.17)	0.84(0.64,1.09)	0.78(0.58,1.05)	0.78(0.59,1.05)	0.61	0.96(0.79,1.17)	0.70

The dietary intake of flavonoids was categorized into five quintiles and tests for trend (p–trend) based on variable containing the median value for each quintile. ln-transformed flavonoids also were utilized as continuous variables for linear regression and *p*-value was used to test significance. Bold indicated statistically significant

Model 1 was a crude model with no adjusted covariates; Model 2 was adjusted for sex, age, race/ethnicity, poverty status, education, smoking status, alcohol consumption, and BMI; Model 3 was further adjusted for hyperlipidemia, CKD, DM, and hypertension based on Model 2

**Fig. 2:**
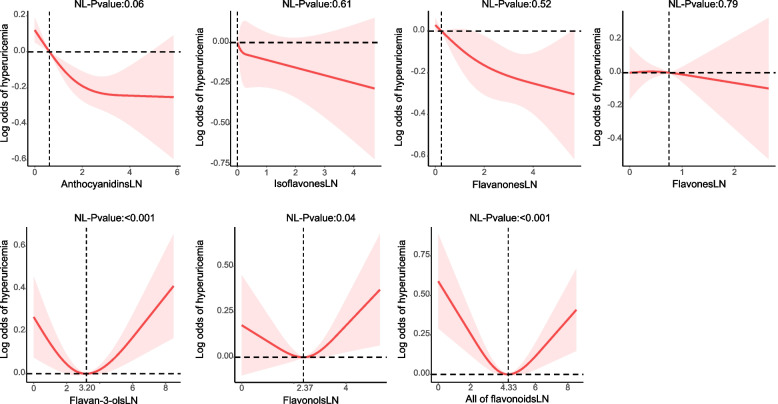
Dose–response associations of flavonoid intake with hyperuricemia. Adjusted for sex, age, race/ethnicity, poverty status, education, smoking status, alcohol consumption, BMI, hyperlipidemia, CKD, DM, and hypertension. P for non-linear < 0.05 was regarded as statistically significant

Figure 2 shows that there is no significant nonlinear association between anthocyanidins, isoflavones, flavanones, or flavones and hyperuricemia in Model 3 based on the RCS analysis. Conversely, in Model 3, it was determined that flavan-3-ols, flavonols, and all flavonoids exhibited a statistically significant nonlinear association with the likelihood of hyperuricemia, displaying a U-shaped pattern (*p*-value for nonlinear: < 0.001, 0.04, and < 0.001, correspondingly). The lowest peak values for flavan-3-ols, flavonols, and all flavonoids were 3.20, 2.37, and 4.33, corresponding to 24.53 mg/100 g foods/day for flavan-3-ols, 10.70 mg/100 g foods/day for flavonols, and 75.94 mg/100 g foods/day for all flavonoids, separately.

## Discussion

As far as we know, this is the first study utilizing a nationally representative sample to reveal the relationship between dietary flavonoid intake and hyperuricemia in U.S. adults. Previous studies have demonstrated a correlation between serum uric acid levels and a variety of chronic diseases [1], and lifestyle habits such as smoking and drinking can also affect the prevalence of hyperuricemia [25]; therefore, we establish three models with progressively increasing inclusion of covariates in linear regression and logistic regression. Although each model accounts for different scenarios, the results of Model 3 are believed to be the most representative of the actual situation. This cross-sectional study uncovered a significant inverse relationship between the intake of anthocyanidins and flavanones and serum uric acid levels in U.S. adults. Additionally, it was found that consuming higher levels of anthocyanins and flavanones was significantly correlated with a decreased incidence of hyperuricemia. A potential nonlinear relationship was observed between the intake of flavan-3-ols, flavonols, and all flavonoids and the incidence of hyperuricemia.

Flavonoids are a class of organic compounds comprising two aromatic carbon rings, namely benzopyran (consisting of the A and C rings) and benzene (comprising the B ring). Anthocyanins and flavones are derived from the basic flavonoid structure. Anthocyanins, including cyanidin, delphinidin, malvidin, pelargonidin, peonidin, and petunidin, are derived mainly from red wine and berries. Flavones, such as apigenin and luteolin, are present in parsley and celery [12]. In line with our findings, administration of single oral anthocyanin extracts was shown to be effective in significantly reducing serum uric acid levels in animal studies [26]. Moreover, previous research has demonstrated that luteolin [27] and apigenin [28] consumption could decrease uric acid levels in animal models of hyperuricemia. Nonetheless, further large population-based studies are needed to confirm the connection of anthocyanins and flavones consumption with serum uric acid levels as well as hyperuricemia incidence.

Tea, wine, beer, citrus fruits, and apples were the most significant sources of flavonoids for U.S. adults. The average total flavonoid intake of U.S. adults was estimated to be 344.8 mg/d [29]. In addition, other studies have estimated total flavonoid intake to range from 200.1 to 445.1 mg/d [30, 31]. Our study shows that the total daily intake of flavonoids was 250.61 ± 10.67 mg/d, with the majority of that coming from flavan-3-ols, which accounted for 201.59 ± 10.07 mg/d. Differences between studies may be related to calculation methods, flavonoid database versions, and population. Our study found that the hyperuricemic group consumed a significantly greater quantity of total flavonoids compared to the non-hyperuricemic group. This is likely because hyperuricemic individuals consume more flavan-3-ols, which are consumed in notably higher quantities than other subclasses. The higher consumption of flavan-3-ols therefore has a significant impact on the total intake of flavonoids. This may help to explain the analogous non-linear association between all flavonoids and flavan-3-ols and hyperuricemia. This non-linear association may explain why no significant association was found between total flavonoid intake and serum uric acid and hyperuricemia. The U-shaped relationship between the odds of hyperuricemia and total flavonoids levels implies that there is an optimal range for flavonoids consumption which is associated with lower hyperuricemia incidence. Additionally, our linear regression model showed a positive correlation between flavonols and serum uric acid. We also observed a non-linear relationship between flavonols and odds of hyperuricemia, suggesting a trend of staging changes of incidence of hyperuricemia with flavonols intake. Additional clinical studies are, of course, needed to verify our findings.

Hyperuricemia can occur due to increased production of uric acid or impaired excretion of uric acid. Currently, the primary treatment for hyperuricemia involves limiting the production of uric acid in the blood, increasing its excretion, and facilitating its dissolution [32]. Purine-rich foods like shellfish, red meat, beer, and sugary beverages raise serum urate levels [1]. Dietary patterns have a significant impact on the prevalence of hyperuricemia. Plant-based diets, such as vegetables, grains, fruits, legumes, and nuts, reduce the risk of hyperuricemia and gout [33]. The effect of the plant-based diet is largely due to the decreased intake of purines in the diet and partly attributable to the selected protective components, which aid in lowering serum uric acid levels. Fruits, vegetables, and other herbs are rich in several micronutrient components that have been found to suppress uric acid formation and are therefore regarded as alternative or supplementary medications for the treatment of hyperuricemia and gout [34]. The prevalence of hyperuricemia was positively correlated with a higher dietary inflammatory index (DII) score in a cross-sectional study conducted in China, indicating that cereals, vegetables, fruits, and other diets with low levels of inflammatory markers may protect against hyperuricemia [35].

The exact mechanism that underlies the association between flavonoid intake and hyperuricemia risk is not yet fully understood. Based on our identification of a significant inverse correlation between the consumption of anthocyanins and flavones and the incidence of hyperuricemia, we have compiled the available evidence and put forth several possible theories. Firstly, flavonoids inhibited xanthine oxidase (XO) and improved renal uric acid excretion. XO played a crucial role in the synthesis of uric acid. Under the catalysis of xanthine oxidase, hypoxanthine was oxidized to xanthine, which was then oxidized further to uric acid [35]. Inadequate renal urate excretion was an additional cause of hyperuricemia. Transporters in the kidneys, such as organic anion transporter 1 (OAT1) and glucose transporter 9 (GLUT9), were responsible for uric acid excretion and reabsorption. It is believed that anthocyanins can hinder XO activity and GLUT9 expression while endorsing OAT1 expression, resulting in an effective pathway for targeting hyperuricemia [36]. In addition, through their anti-inflammatory characteristics, flavonoids could reduce hyperuricemia. Luteolin, a flavone compound, might reduce the uric acid content in rat liver tissue by lowering tumor necrosis factor (TNF)-a, interleukin (IL)-1β, and IL-6 levels [27]. Both anthocyanins and flavones were demonstrated notable inhibitory effects on NLRP3 inflammasomes [13]. The increases in cellular uric acid resulting from oxidative stress mediated by XO may be related to the NLRP3 inflammasome [37]. NLRP3 inflammasome was also crucial to the development of hyperuricemic nephropathy [38]. Therefore, it was vital to clarify the effect of flavonoid supplementation on serum uric acid and avoid hyperuricemia from occurring.

The strengths of this study are as follows. This was the first study to examine the relationship between flavonoids in the diet and the risk of hyperuricemia using a large sample of U.S. adults (8,760 individuals) and a national sample. Second, we adjusted for numerous confounding variables and established three distinct models for analysis. However, our research still had some limitations. Since the amount of flavan-3-ols was significantly greater than that of other subclasses of flavonoids, our conclusions about the correlation between all flavonoid intake and hyperuricemia may be biased. In addition, there were limitations in obtaining data, which prevented us from incorporating other diets that are associated with serum uric acid levels, including seafood and beer, into the correction model. Moreover, it is important to acknowledge that the estimation of daily flavonoid intake was based on only two days of dietary data, which might have limited the accuracy and representativeness of the results. Finally, since our study was cross-sectional, additional prospective longitudinal research and clinical trials were necessary to corroborate these results.

## Conclusion

Our study demonstrated that individuals who follow a diet rich in anthocyanins and flavanones had significantly lower serum uric acid levels and a lower incidence of hyperuricemia. For the general population, consumption of anthocyanins- and flavanones-rich foods may associate with a lower risk of hyperuricemia, which could be useful in developing strategies aimed at preventing hyperuricemia.

## Supplementary Information

**Additional file 1:** **Supplementary Table 1. **29 flavonoids in 6 flavonoid classes. **Supplementary Table 2. **Quintile values of flavonoid.

## Data Availability

More information about the NHANES could be obtained at: http://www.cdc.gov/nhanes.

## Abbreviations

NHANES: National Health and Nutrition Examination Survey
RCS: Restricted cubic splines
BMI: Body mass index
CKD: Chronic kidney disease
DM: Diabetes mellitus
USDA: United States Department of Agriculture
NCHS: National Center for Health Statistics
MEC: Mobile examination center
FNDDS: Food and Nutrient Database for Dietary Studies
PIR: Poverty income ratio
ATP3: Adult Treatment Panel III
TG: Triglycerides
TC: Total cholesterol
LDL: Low-density lipoprotein
HDL: High-density lipoprotein
ACR: Albumin-to-creatinine ratio
OGTT: Oral glucose tolerance test
BP: Blood pressure
ORs: Odds ratios
CIs: Confidence intervals
DII: Dietary inflammatory index
XO: Xanthine oxidase
OAT1: Organic anion transporter 1
GLUT9: Glucose transporter 9
TNF: Tumor necrosis factor
IL: Interleukin
