# Supplementary material for: Association between dietary intake of flavonoids and hyperuricemia: a cross-sectional study
Source: BMC Public Health. 2023 Jun 24;23:1227. doi: 10.1186/s12889-023-16134-4 (PMC10290396; doi:10.1186/s12889-023-16134-4)
Supplement: Supplementary file 1 — Additional file 1: Supplementary Table 1. 29 flavonoids in 6 flavonoid classes. Supplementary Table 2. Quintile values of flavonoid. [file 12889_2023_16134_MOESM1_ESM.docx]

**Supplementary materials**

**Supplementary Table 1.** 29 flavonoids in 6 flavonoid classes.

| All 29 Flavonoids | |
| --- | --- |
| Anthocyanidins | Cyanidin |
|  | Delphinidin |
|  | Malvidin |
|  | Pelargonidin |
|  | Peonidin |
|  | Petunidin |
| Flavan-3-ols | Epicatechin |
|  | Epicatechin 3-gallate |
|  | Epigallocatechin |
|  | Epigallocatechin-3-gallate |
|  | Catechin |
|  | Gallocatechin |
|  | Theaflavin |
|  | Theaflavin-3-3-digallate |
|  | Theaflavin-3q-gallate |
|  | Theaflavin-3-gallate |
|  | Thearubigins |
| Flavanones | Eriodictyol |
|  | Hesperetin |
|  | Naringenin |
| Flavones | Apigenin |
|  | Luteolin |
| Flavonols | Isorhamnetin |
|  | Kaempferol |
|  | Myricetin |
|  | Quercetin |
| Isoflavones | Daidzein |
|  | Genistein |
|  | Glycitein |
|  | Subtota-Catechins |

**Supplementary Table 2.** Quintile values of flavonoid.

| Variable | Quintile of flavonoids (mg and n%) | | | | |
| --- | --- | --- | --- | --- | --- |
|  | Q1 | Q2 | Q3 | Q4 | Q5 |
| Isoflavones | (,0] 59.27% | / | (0,0.01] 5.66% | (0.01,0.07] 16.42% | (0.07,) 18.65% |
| Anthocyanidins | (,0] 34.16% | (0,0.1] 5.97% | (0.1,2.27] 19.87% | (2.27,10.45] 20.02% | (10.45,)19.98% |
| Flavan-3-ols | (,1.82] 20% | (1.82,7.38] 20.05% | (7.38,17.67]19.98% | (17.67,232.54]19.98% | (232.54,) 20% |
| Flavanones | (,0] 36.15% | (0,0.07] 4.08% | (0.07,0.65] 19.82% | (0.65,20.46] 19.95% | (20.46,) 20% |
| Flavones | (,0.05] 20.31% | (0.05,0.26] 19.89% | (0.26,0.61] 19.92% | (0.61,1.28] 20% | (1.28,) 19.89% |
| Flavonols | (,4.67] 20.03% | (4.67,9.39] 19.97% | (9.39,16.04]20.02% | (16.04,28.11]19.98% | (28.11,) 20% |
| All of flavonoids | (,13.92]20.0% | (13.92,33.35]19.9% | (33.35,72.702] 20% | (72.70,310.22] 20% | (310.22,) 20% |
